# A Digitally Guided, Single-Visit Workflow for Miniscrew-Assisted Rapid Palatal Expansion: The YOM Technique

**DOI:** 10.7759/cureus.115777

**Published:** 2026-09-04

**Authors:** Mohammed Yasser Alshamly, Moustafa M. A. Abdelhamid, Mohamed A Marei

**Affiliations:** 1 Orthodontics, Ministry of Health and Population, Alexandria, EGY

**Keywords:** digital orthodontics, marpe, mid palatal suture, palatal expansion, palatal miniscrew

## Abstract

Miniscrew-assisted rapid palatal expansion (MARPE) extends non-surgical, predominantly skeletal expansion to skeletally mature patients. Its success depends on the palatal miniscrews achieving sound engagement in a safe site and on the expander and the screws fitting together accurately. Two sequences are described. In a bone-first sequence, the miniscrews are inserted first, commonly through a CAD/CAM (computer-aided design/computer-aided manufacturing) guide that controls trajectory and depth, after which the appliance must be fitted to the screws in the positions they actually occupy. In an appliance-first sequence, the expander is cemented first, and the miniscrews are inserted through its own holes, so the entry position is defined by the appliance while angulation and depth are left to the operator. This technical report describes the YOM technique, an appliance-first workflow in which guided insertion is applied. A single registered plan of superimposed cone-beam CT (CBCT) and intraoral scan data is used to design a metal-printed hybrid tooth- and bone-borne MARPE appliance together with a separate, removable surgical guide. The appliance is cemented first, and the guide is then indexed onto it so that its guide holes are coaxial with the appliance's miniscrew holes. The guide is intended to control the entry site, angulation, and insertion depth. Because the appliance is cemented before insertion, the screws need not be parallel, and each trajectory can be planned according to the bone available at that site. Appliance delivery and guided miniscrew insertion are completed at a single clinical appointment, without the appliance having to be adapted to the screws afterwards. The individual components of the workflow are established in the literature; what is described here is their integration. No patient data are reported, and the insertion accuracy achieved with this guide has not been compared with the plan.

## Introduction

Transverse maxillary deficiency commonly presents as a unilateral or bilateral posterior crossbite, a narrow maxillary arch, dental crowding, and wide buccal corridors [1]. In growing patients, tooth-borne rapid maxillary expansion readily corrects this deficiency by opening the midpalatal suture. As skeletal maturity increases, the suture becomes progressively interdigitated and then fused, and the response shifts: a growing share of the arch-width change comes from buccal tipping and dentoalveolar bending of the posterior teeth rather than from true separation of the suture [2,3]. Clinically, this presents as tipping of the posterior crowns, buccal bone dehiscence, and gingival recession, and in advanced stages of maturation, tooth-borne expansion may fail to open the suture at all. Because chronological age is an unreliable indicator of suture status, Angelieri and coworkers introduced a cone-beam CT (CBCT) classification of midpalatal suture maturation in five stages (A-E) [4]. This individualized assessment guides the choice between conventional expansion, miniscrew-assisted expansion, and surgical approaches.

Miniscrew-assisted rapid palatal expansion (MARPE) extends non-surgical, predominantly skeletal expansion to skeletally mature adolescents and adults. Because the expansion force is transmitted to the basal bone through palatal miniscrews rather than through the teeth, MARPE produces a larger skeletal and a smaller dentoalveolar component than tooth-borne expansion, with less buccal tipping and less buccal bone loss [5,6]. A meta-analysis of eight observational studies reported a pooled success rate of 92.5% (95% confidence interval (CI): 88.7-96.3%) in patients aged 16 years and older, although the authors graded the certainty of this evidence as very low [5].

The clinical success of MARPE depends on the palatal miniscrews achieving sound, ideally bicortical, engagement in a safe site, and on the expander seating passively on parallel, well-positioned screws [7]. Two sequencing concepts are described in the literature [8]. In the “bone-first” sequence, the miniscrews are placed first, commonly through a CAD/CAM (computer-aided design/computer-aided manufacturing) insertion guide that controls the trajectory and insertion depth, and the expander is then fitted to the screws as placed [9-12]. In the “appliance-first” sequence, the expander is cemented first, and the miniscrews are inserted through the appliance’s own holes, so that the cemented appliance itself acts as the surgical guide [13-17].

Each sequence carries a different limitation. In the bone-first sequence, the guide governs the insertion, and the appliance must subsequently be fitted to the screws in the positions they actually occupy; where the achieved positions differ from the plan, the appliance may need to be adapted to them, and in several published protocols the appliance is therefore fitted or delivered after a further set of records [9-12]. In the appliance-first sequence, the screws and the expander are delivered together, but the appliance’s holes constrain only the entry position: they do not control angulation or insertion depth, and the site itself is dictated by the appliance rather than by the bone. Wilmes has stated this second objection explicitly, noting that with the conventional appliance-first method the prefabricated nature of the appliance restricts the placement site and prevents the operator from choosing that site freely based on bone quality [8]. Notably, insertion guides have been described only in combination with the bone-first sequence [9-12].

This article describes a workflow developed by the authors, the YOM technique, which applies guided insertion to the appliance-first sequence rather than to the bone-first sequence. A single registered digital plan produces both a metal-printed hybrid tooth- and bone-borne MARPE appliance and a separate, removable surgical guide that seats on that appliance once it is cemented; the same workflow can also be used with bone-borne MARPE, so that the surgical guide holes are coaxial with the appliance’s miniscrew holes. The miniscrews are therefore inserted with planned control of the insertion site, angulation, and depth through an expander that is already in place, and no adaptation of the appliance to the screws is required afterwards. Throughout this report, “same appointment” refers to delivery of the appliance together with insertion of the miniscrews at one clinical visit; expansion and retention necessarily continue over the following weeks and months. The workflow is described step by step; no patient data are reported. (The name YOM is formed from the names of the authors who developed the technique.)

## Technical report

The YOM technique

The defining feature of the YOM technique is that one digital plan generates both the expander and the surgical guide, and that the guide indexes onto the cemented expander rather than onto the dentition alone. The workflow proceeds in six steps.

Step 1. Digital Records

A CBCT and an intraoral scan are obtained and imported into the planning software (OnyxCeph³™, Image Instruments GmbH, Chemnitz, Germany; www.onyxceph.com). The CBCT provides palatal bone volume, cortical thickness, the position of the nasal floor and the roots, and the midpalatal suture status. The intraoral scan provides an accurate surface model of the dentition and palate for appliance design.

Step 2. Virtual Planning of the Miniscrews

The CBCT and the intraoral scan are superimposed. The superimposed data set allows the operator to assess the anatomy of the region before any screw is placed: the availability and thickness of palatal bone, the position and contour of the nasal floor, the roots of the adjacent teeth and the interradicular spaces where these are relevant, and any other structure that should be respected. Two paramedian palatal miniscrews are then planned in the anterior palate, in sites selected for adequate bone thickness, sufficient interradicular and nasal clearance, and, where possible, bicortical engagement. The two screws need not be parallel: because the appliance is cemented before insertion and each screw passes through its own hole, the appliance is never seated over both screws at once. Each trajectory can therefore be planned independently, within the tolerance of its appliance hole, for the bone available at that site (Figure 1). Because the screw sites are chosen on the CBCT before the appliance is designed, site selection is driven by the bone rather than by a prefabricated appliance.

**Figure 1:**
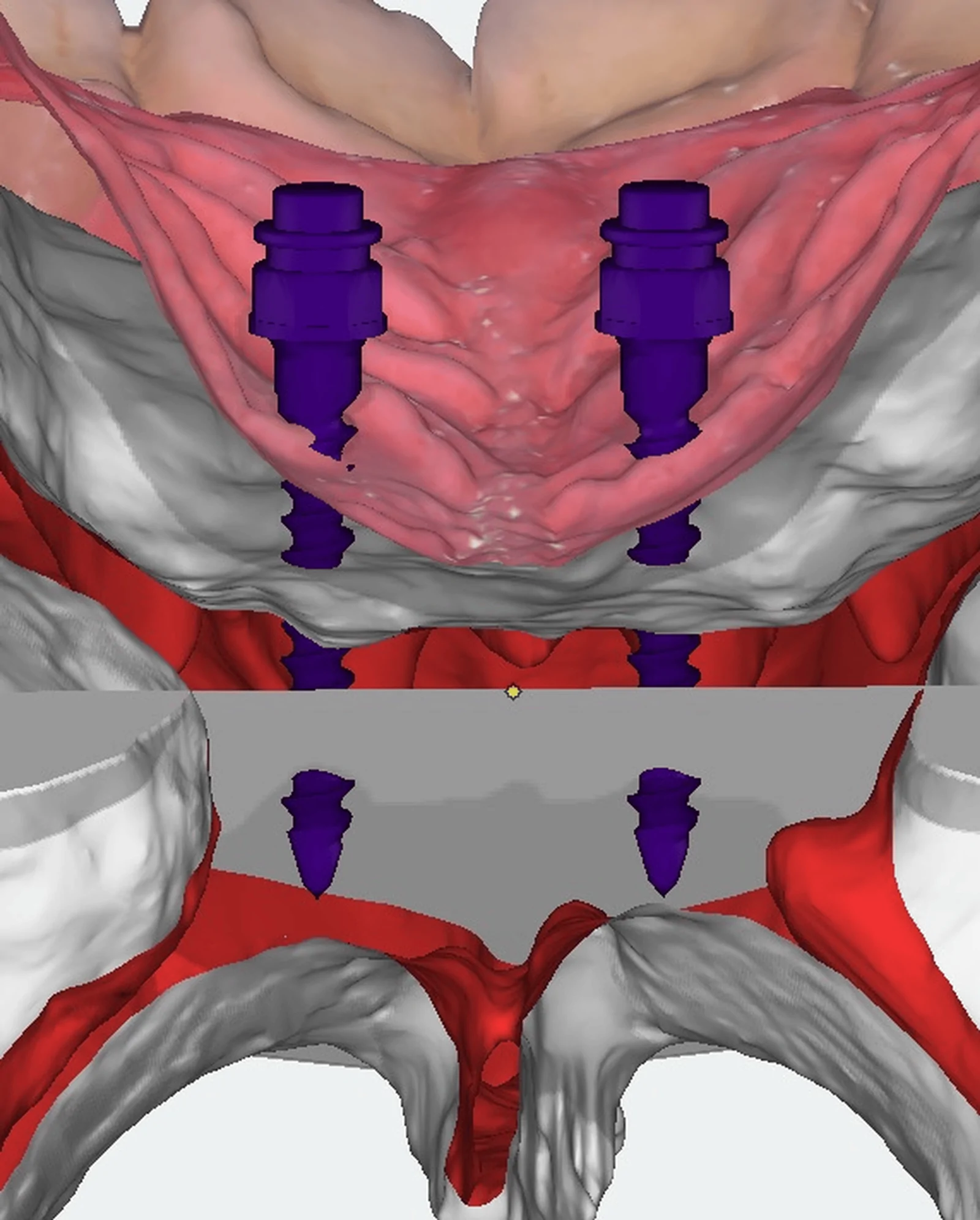
Virtual planning of the two paramedian palatal miniscrews Step 2 of the workflow. Planned palatal miniscrew positions on the superimposed cone-beam CT and intraoral scan, shown relative to the nasal floor, the roots, and the palatal bone CT: computed tomography

Step 3. Combined Appliance and Guide Design

From the same data set, a hybrid tooth- and bone-borne MARPE appliance is designed: a midline expansion screw connected anteriorly to the two planned paramedian miniscrew positions and posteriorly to shells that seat over the maxillary first molars, so that no conventional molar bands are required. A removable surgical guide is then designed on top of that appliance, so that it is supported on the occlusal and palatal surfaces of the dentition and indexes onto the appliance framework (Figure 2). The surgical guide is made coaxial with the planned screw trajectories and, therefore, with the appliance’s own miniscrew holes. The guide also carries a depth stop, set to the planned insertion depth.

**Figure 2:**
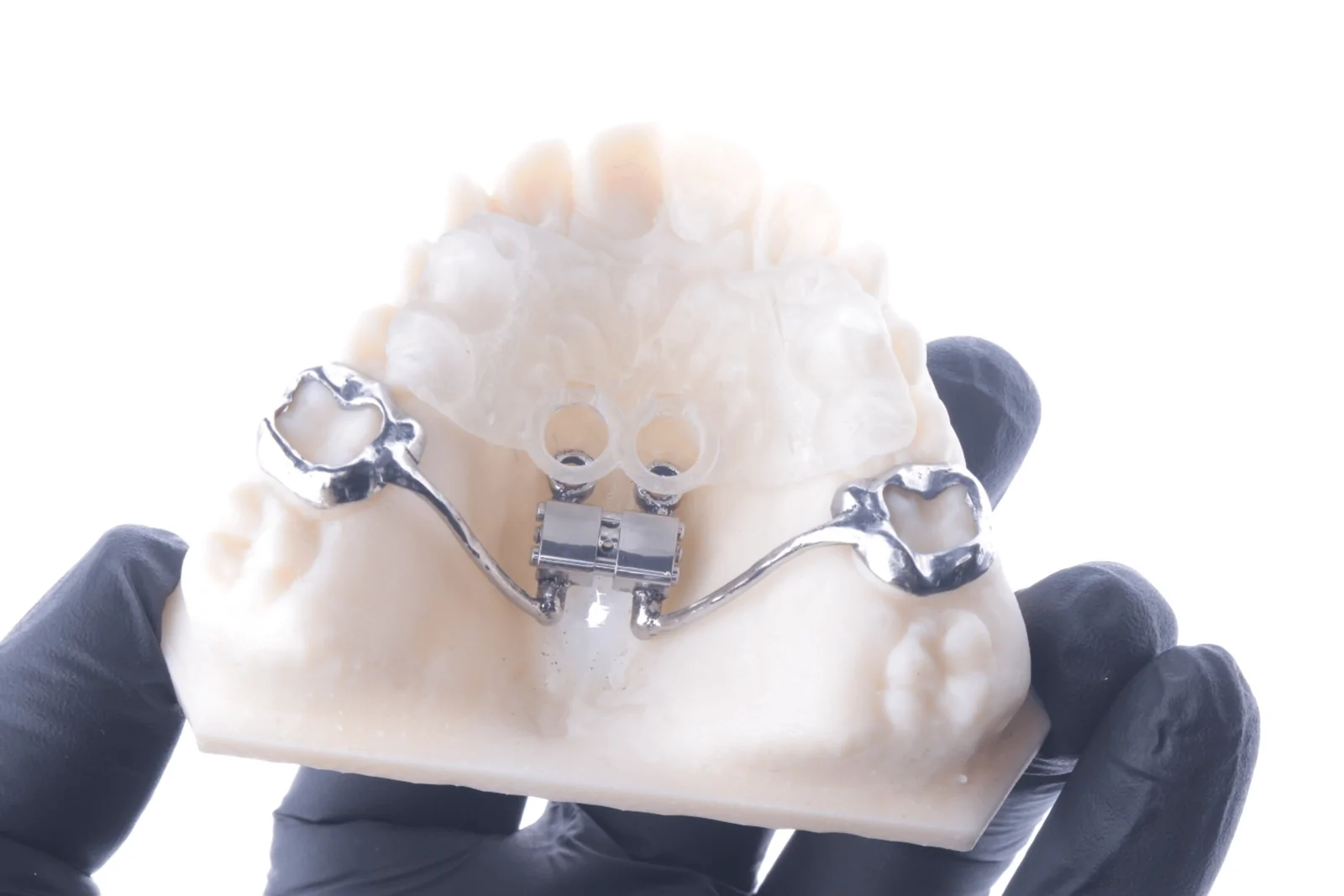
The hybrid expander and the surgical guide produced from a single digital plan Steps 3 and 4 of the workflow. The fabricated hybrid tooth- and bone-borne expander and the 3D-printed surgical guide, shown together on the printed model before delivery

Step 4. Fabrication

The guide is 3D-printed in a biocompatible resin. The hybrid MARPE appliance is manufactured directly from the same digital design by selective laser melting, as a single printed metal unit incorporating the palatal framework and the molar shells, and the midline expansion screw is joined to it. Because both the appliance and the guide are produced from the digital plan, no physical impression, plaster model, or laboratory wire bending and soldering is required at any stage. Both are then sterilized and prepared for the delivery appointment.

Step 5. Same-Appointment Delivery and Guided Insertion

At a single appointment, the hybrid MARPE appliance is first cemented onto the maxillary first molars, through its printed shells, with a dual-cured, self-adhesive resin cement (TheraCem CA, BISCO, Inc., Schaumburg, IL; www.bisco.com), which seats the expander and its miniscrew holes in the planned position. The surgical guide is then seated over the cemented appliance. Because the guide registers on both the appliance and the dentition, it is intended to seat stably and unambiguously, and its holes reproduce the planned trajectories (Figure 3). Under local palatal anesthesia, the two palatal miniscrews (OrthoEasy Pal, FORESTADENT Bernhard Förster GmbH, Pforzheim, Germany; www.forestadent.com) are inserted through the surgical guide and through the appliance’s miniscrew holes, following the planned site, angulation, and depth (Figure 4). Before the guide is removed, the operator should confirm that both screws are fully seated against their depth stops and that the appliance has not been displaced during insertion. The guide is then removed, leaving the expander fixed to its anchorage (Figure 5).

**Figure 3:**
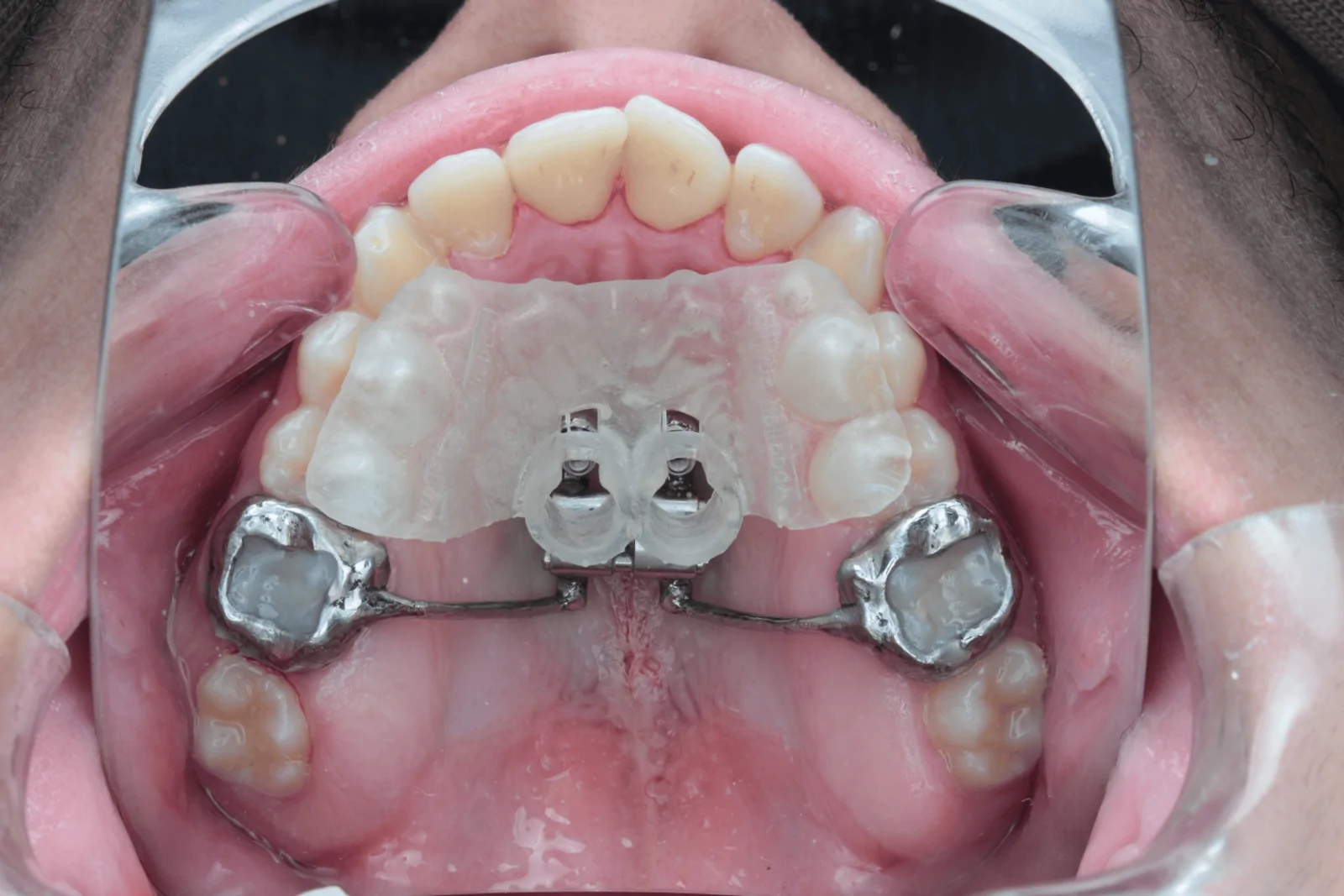
The surgical guide seated over the cemented appliance Step 5 of the workflow. The guide registers on both the cemented appliance and the dentition so that its holes reproduce the planned screw trajectories

**Figure 4:**
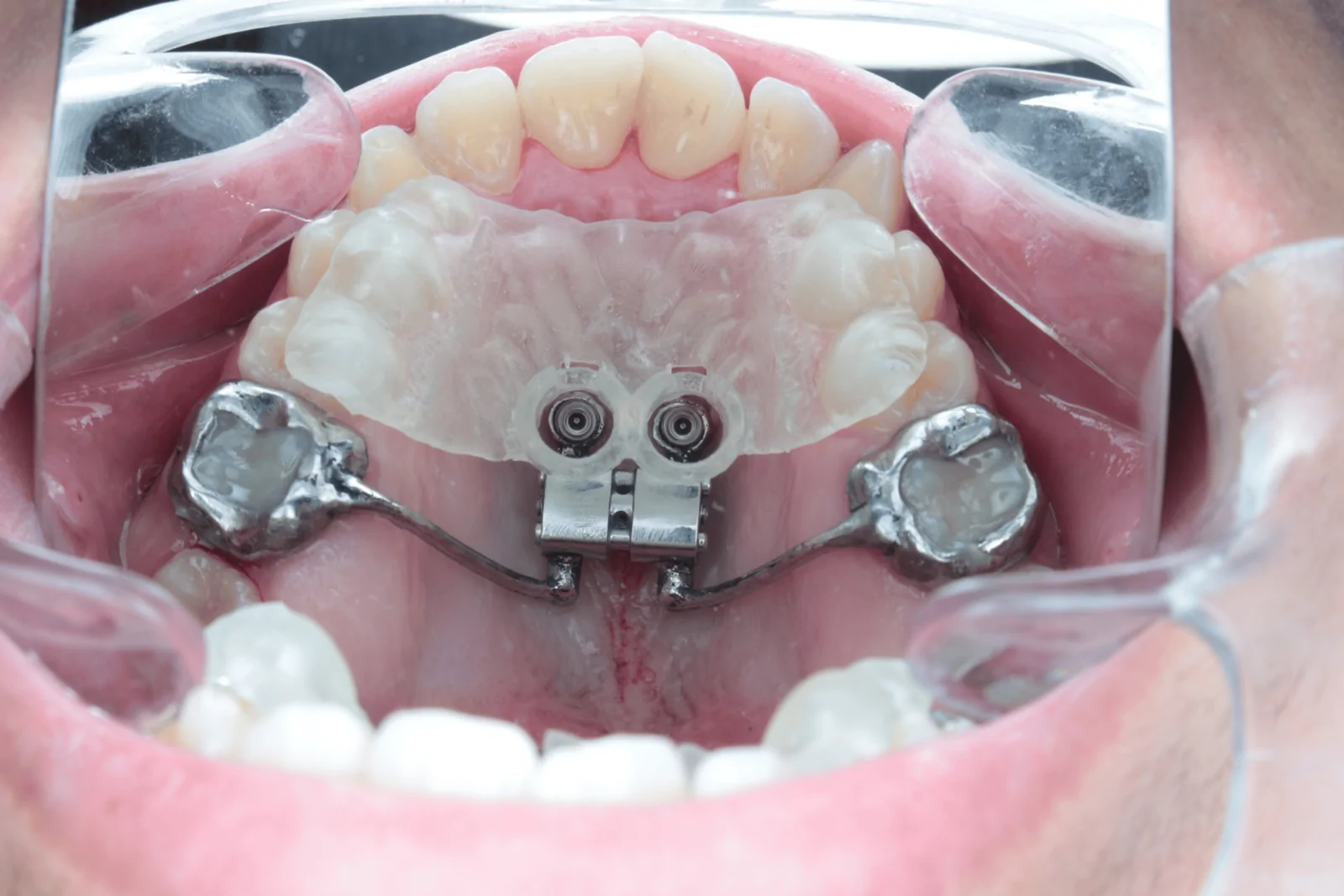
Guided insertion of the palatal miniscrews through the guide sleeves Step 5 of the workflow. The two miniscrews are inserted through the surgical guide and the miniscrew holes of the appliance. The symmetric “owl-eye” appearance confirms both screws engaged through the guide and the appliance in their planned positions

**Figure 5:**
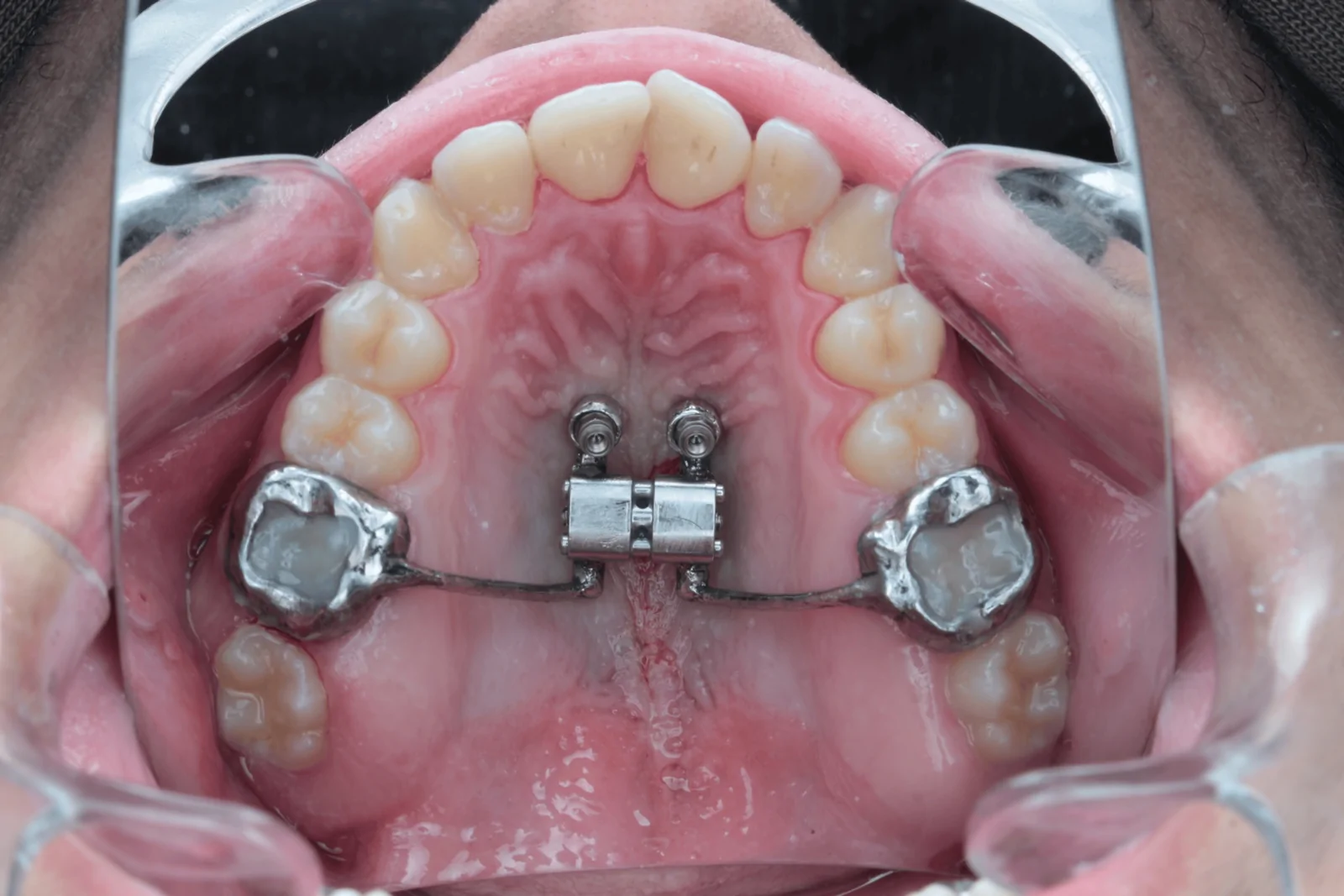
Maxillary occlusal view with the guide removed The hybrid appliance in situ: the midline expansion screw connected to two paramedian palatal miniscrews anteriorly and to bands on the maxillary first molars

The guide is intended to provide control in three respects that the cemented appliance alone cannot. The entry site is defined by the appliance’s miniscrew holes, which reproduce the planned sites; the angulation, or trajectory, is defined by the axis of the corresponding guide holes; and the insertion depth is limited by the depth stop associated with those holes. In conventional appliance-first insertion, only the entry position is constrained, and angulation and depth remain matters of operator judgment. This transfer of all three planned parameters, through an appliance that is already cemented, is the principal technical feature of the workflow.

Step 6. Activation and Retention

The expander is activated according to the operator’s preferred protocol; we use four quarter-turns on the day of delivery and one quarter-turn (approximately 0.2 mm) per day thereafter until the planned expansion is achieved. The appearance of a midline interincisal diastema confirms opening of the midpalatal suture. Once expansion is complete, the appliance is converted into a passive retainer: the molar arms are sectioned, and the expansion screw is locked with flowable composite resin. The correction is then held directly on the skeletal anchorage, and the maxillary molars are freed (Figure 6). The retainer is left in place while the midpalatal suture consolidates. The activation and retention phases extend over weeks and months; only the appliance delivery and the guided insertion take place at the single appointment described in Step 5.

**Figure 6:**
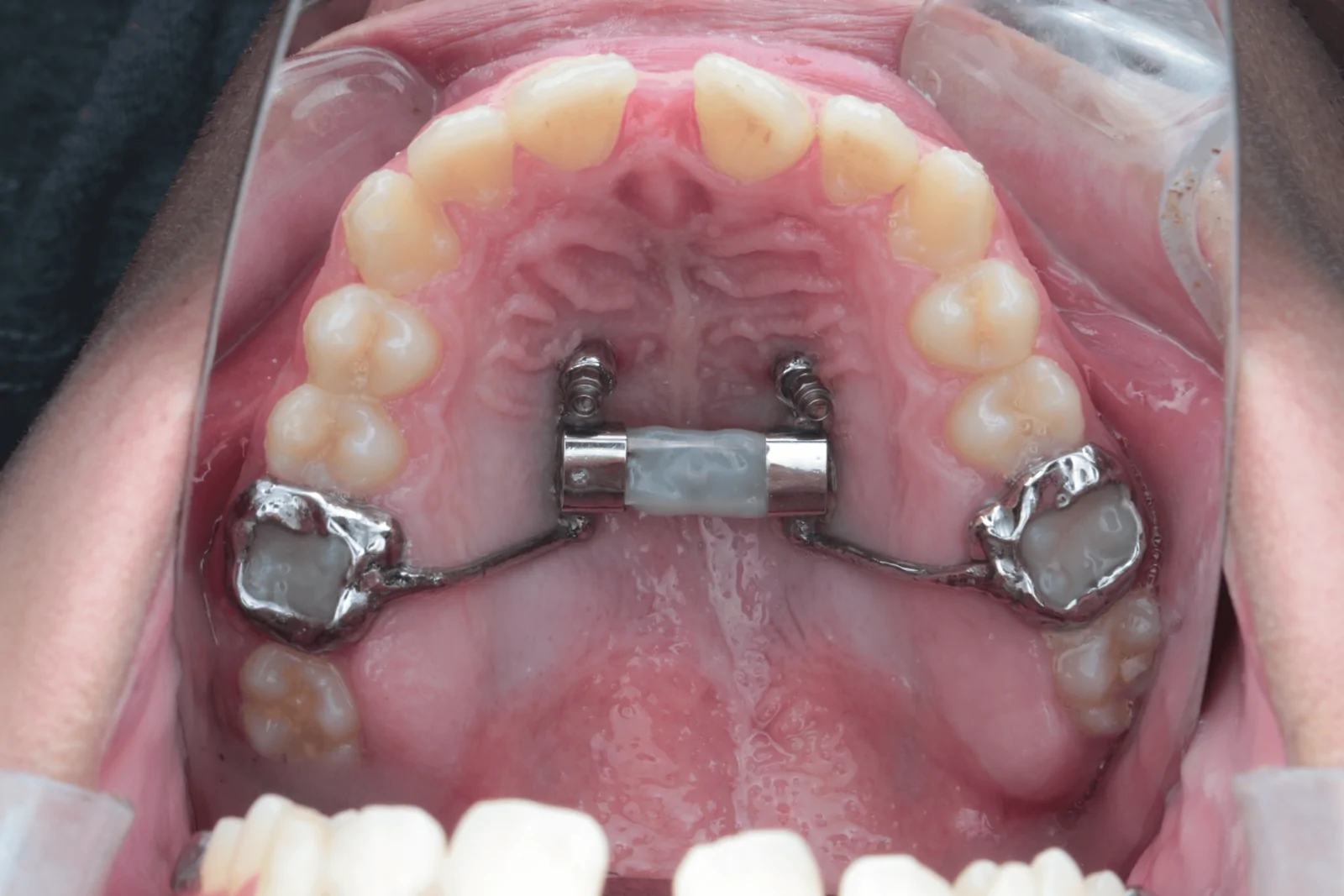
Conversion of the expander into a passive retainer Step 6 of the workflow. The molar arms have been sectioned, leaving the expander retained on the two palatal miniscrews, with the expansion screw locked in flowable composite resin

Verification and documentation

A workflow of this kind should be documented in a way that allows the skeletal component of the correction to be verified rather than assumed. Three points deserve emphasis.

Confirming that the suture has opened. The appearance of a midline interincisal diastema during the first days of activation is the standard clinical sign that the midpalatal suture has separated, and it can be corroborated radiographically on an occlusal radiograph or on an axial CBCT section parallel to the palatal plane. This confirms that separation has occurred; it does not by itself quantify how much of the measured arch-width change is skeletal.

Separating the skeletal from the dental component. Distinguishing skeletal from dentoalveolar expansion requires comparison of pre- and post-expansion CBCT volumes for suture separation and for the buccolingual inclination of the posterior teeth. A post-expansion CBCT acquired while the appliance is still in the mouth is degraded by metal artifact from the expansion screw, the framework, and the miniscrews. That artifact obscures the thin buccal cortical plate and blurs the outline of the crowns and roots used to construct each tooth’s long axis, so inclination values measured under those conditions carry an appreciable and unquantified error. Such measurements should be reported as indicative only. Where the skeletal-to-dental ratio is to be quantified, the confirmatory CBCT should be deferred until after the appliance has been removed, and the measurement protocol should be stated with the scan parameters, the reference planes, and the landmarks used.

Measurement reliability

Any linear or angular measurement reported from these records should be made by a calibrated examiner. The recommended minimum is that a random subsample of measurements be repeated by the same examiner after an interval of at least two weeks, and that a second examiner independently repeat those measurements. Intra- and inter-examiner agreement should then be quantified - for example, by the intraclass correlation coefficient together with Dahlberg’s method error - and reported alongside the values themselves. Measurements derived from a single unrepeated assessment, or from artifact-degraded images, should not be presented as quantitative evidence of a skeletal response.

Relationship to previously described digital MARPE workflows

Digitally guided and customized MARPE has an established literature, and the individual components of the present workflow are not new. Superimposition of CBCT and intraoral scan data to plan palatal miniscrew position, length, and angulation has been standard practice for several years [10,13,15]. Customized MARPE frameworks manufactured from that plan by direct metal laser sintering or selective laser melting have been described repeatedly [14,15,18]. 3D-printed insertion guides with or without sleeves for palatal miniscrews are likewise well established, both for MARPE and for palatal anchorage devices generally [9,11,12,19]. Single-appointment delivery of miniscrews together with a maxillary expander was reported by De Gabriele and coworkers in 2017 [9], and the use of one registered plan to produce both a printed guide and a printed appliance has since been described for bone-first workflows [18,19]. The appliance-first principle, in which the cemented expander itself acts as the surgical guide, has also been described on several occasions [13-17].

Table 1 sets out these reports against four characteristics: whether the miniscrew positions were digitally planned, whether a separate physical insertion guide was used intraorally, whether the appliance was produced from the same digital plan, and whether the screws or the appliance were delivered first. Read together, the prior art falls into two groups. In the first, a guide controls trajectory and depth, but the screws are placed before the appliance, which must then be related and, if necessary, adapted to them [9,11,12,19]. In the second, the appliance is cemented first and guides the screws through its own holes, which fixes the entry position but leaves angulation and depth to the operator’s judgment [13,15-17]. The closest reported intermediate is the customized framework of Cantarella and coworkers, in which guiding bushings were built into the appliance itself by selective laser melting [14].

**Table 1:** Comparison of previously described digitally guided and customized MARPE workflows with the technique described here Superscript numbers refer to the reference list. "Bone first" denotes miniscrew insertion before appliance delivery; "appliance first" denotes cementation of the expander before the miniscrews are inserted through it MARPE: miniscrew-assisted rapid palatal expansion; MSE: maxillary skeletal expander; SLM: selective laser melting; TAD: temporary anchorage device

Report	Miniscrew positions digitally planned	Separate insertion guide used intraorally	Appliance produced from the same digital plan	Sequence of delivery
De Gabriele et al., 2017 [9]	Yes	Yes (printed insertion guide)	Semi-customized (hybrid Hyrax)	Bone first; screws then expander, one appointment
Minervino et al., 2019 [12]	Yes	Yes	No (stock MARPE, included in the plan)	Guided insertion in a single session
Cantarella et al., 2020 [13]	Yes	No (laboratory positioning guide only)	No (conventional fabrication on the plan)	Appliance first; cemented expander acts as the guide
Thakkar et al., 2020 [15]	Yes	No	Yes (direct metal laser sintering)	Appliance first; screws through the appliance holes
Cantarella et al., 2021 [14]	Yes	No (guiding bushings built into the appliance)	Yes (selective laser melting)	Appliance first; screws through slots and bushings
Wilmes et al., 2022 [10]	Yes	Yes (printed insertion guide)	Semi-customized (Quadexpander)	Bone first; expander fixed to the screws, one appointment
Wilmes et al., 2022 [11]	Yes	Yes, removable guide	Semi-customized (Quadexpander)	Bone first; expander attached at the same appointment
Ludwig et al., 2022 [18]	Yes	Yes (printed insertion guide)	Yes (selective laser melting)	One session; MARPE and screws installed together, order not stated
Pozzan et al., 2022 [19]	Yes	Yes, removable guide	Yes (designed on the planned screw positions)	Bone first; one-visit protocol (palatal TAD appliance)
Hsu et al., 2023 [16]	Yes	No (transfer index used on the model)	No (stock MSE II)	Appliance first; cemented expander acts as the guide
Andrei et al., 2025 [17]	Yes	No	Yes (resin modelling or additive manufacture)	Appliance first; screws through the appliance insertion sites
The YOM technique (present report)	Yes	Yes — removable guide indexed onto the cemented appliance, with depth stops	Yes — metal-printed (SLM) framework with molar shells, no bands; appliance and guide from one registered plan	Appliance first; guided screws through the appliance holes, one appointment

The contribution of the present workflow is therefore an integration rather than a new component. We could not identify a published description of a separate, removable surgical guide that indexes onto an already-cemented hybrid MARPE appliance so that its holes are coaxial with the appliance’s miniscrew holes. The elements that are combined here are a digitally planned hybrid MARPE appliance; a separate removable surgical guide produced from the same registered plan; the indexing of that guide onto the appliance after cementation; the coaxial relationship between the guide and the appliance’s miniscrew holes; guided control of trajectory and insertion depth; the appliance-first sequence; and delivery of appliance and screws at one appointment.

The arrangement also addresses the objection to appliance-first placement raised by Wilmes, since the screw sites are selected on the CBCT before the appliance is designed rather than being dictated by a prefabricated framework [8]. We make no claim of priority for digital planning, for CBCT and intraoral scan superimposition, for customized appliance manufacture, for printed insertion guides, for guide sleeves, or for single-appointment delivery, each of which is credited above. Nor do we claim priority for manufacturing the appliance rather than bending and soldering it in the laboratory: metal-printed MARPE frameworks and printed molar shells in place of conventional bands are both established in CAD-CAM palatal appliance workflows [20,21].

## Discussion

The rationale for guiding the insertion rests on a consistent body of accuracy data. In a systematic review and meta-analysis of interradicular miniscrew placement, static computer-assisted insertion was associated with less iatrogenic root damage than freehand placement (0.6% versus 21.4% of miniscrews), with angular deviations of 1.8-4.7° for guided and 6.3-9.6° for freehand insertion [22]. In the palate specifically, fully digitally planned insertion guides allowed accurate placement of miniscrews in a cadaver model [23]. A subsequent cadaver study of simultaneous miniscrew placement and appliance fitting reported the lowest mean angular deviation with a fully digital workflow and concluded that such a workflow may be advantageous because it requires only a single visit [24]. The palate is a site where this matters: the nasal floor must be respected, and bicortical engagement is desirable where the anatomy allows.

In a bone-first sequence, the two screws must also be close to parallel, because the appliance is subsequently seated onto them. In the present workflow, that constraint is relaxed: the appliance is already cemented, and each miniscrew is inserted through its own hole rather than the appliance being seated over both screws. Each trajectory can therefore be planned independently, within the tolerance of its appliance hole, for the bone available at that site. Digital planning allows these structures to be identified and a trajectory selected in relation to them, and guided insertion is intended to transfer that planned trajectory and depth to the patient. It does not guarantee that any anatomical structure will be avoided; the guide transfers a plan, and the quality of the result still depends on the quality of the plan and on the execution.

The workflow is intended to offer the following practical advantages. First, appliance delivery and guided insertion take place at one appointment, which reduces the number of visits compared with the frequently and more commonly used bone-first two-visit workflow in which the appliance is fitted after the screws. Second, because the appliance is cemented before the miniscrews are inserted and the guide is indexed directly to the cemented appliance, the sequence is not intended to require any subsequent adaptation of the appliance to the achieved screw positions; this is a rationale for the sequence rather than a demonstrated clinical superiority. Third, the screw trajectory is pre-planned on the anatomy rather than estimated freehand, and the depth is limited by a stop. Fourth, the interval between insertion and the start of activation is shortened. Fifth, the choice of screw site remains with the clinician and the CBCT rather than being determined by the appliance geometry.

A further consideration concerns the miniscrews themselves. Some bone-first and customized workflows use a miniscrew of a particular design that is compatible with the appliance’s attachment or abutment system, and such miniscrews may be more expensive than conventional orthodontic miniscrews. Because the YOM appliance is designed around its own miniscrew holes on the basis of the screw selected at the planning stage, the workflow may allow that palatal miniscrew to be used without requiring a proprietary abutment design. We have not compared costs, and we do not suggest that the workflow is compatible with every conventional miniscrew; this is offered as a potential practical and economic consideration rather than a demonstrated saving.

When any guided workflow is evaluated, several distinct sources of deviation should be distinguished: the manufacturing accuracy of the guide, the manufacturing accuracy of the appliance, the seating of the appliance and of the guide in the mouth, and the accuracy of the insertion itself. Manufacturing tolerances or imprecise seating of either component may contribute to a discrepancy between the planned and the achieved miniscrew position. We do not suggest that such a discrepancy necessarily produces a clinically significant seating failure, and the extent to which these sources contribute in this workflow has not been established.

Several limitations should be stated. This report describes a technique; it presents no clinical series and no comparative data, and it therefore cannot establish efficacy, accuracy, or any advantage over existing MARPE workflows. The accuracy of the insertions achieved with this guide has not been quantified against the plan. Doing so is the necessary next step before the workflow can be recommended over established alternatives, and future studies should compare the planned and achieved entry point, the planned and achieved apex position, the angular deviation, and the depth deviation of each miniscrew, together with chair time, miniscrew survival, and the seating accuracy of the appliance and of the guide.

The workflow requires a CBCT, an intraoral scan, planning software, and digital fabrication, all of which add cost, require a learning curve, and carry the corresponding radiation and resource considerations. Its accuracy depends on stable, unambiguous seating of the guide on the cemented appliance, which may be more difficult where palatal bone volume is limited, where the appliance has been displaced during cementation, or where restorations or a shallow palatal vault compromise the support surfaces. Finally, the approach is applicable only where the midpalatal suture is not so far fused that non-surgical expansion would be expected to fail.

## Conclusions

The YOM technique is a digital workflow in which a single registered plan produces both a metal-printed hybrid MARPE appliance and a separate removable surgical guide that indexes onto that appliance once it has been cemented. It applies guided, depth-controlled miniscrew insertion to the appliance-first sequence, so that the expander and its skeletal anchorage are delivered at one appointment, and the appliance does not have to be adapted to the screws afterwards. The individual elements of the workflow are drawn from established practice; the integration described here appears not to have been reported previously. Its accuracy and any clinical advantage remain to be quantified in comparative studies.
